# Population-based human biomonitoring in the ‘Land of Fires’ area: innovations in study design and procedures

**DOI:** 10.2144/fsoa-2020-0164

**Published:** 2020-10-22

**Authors:** Biancamaria Pierri, Carlo Buonerba, Annachiara Coppola, Antonio Pizzolante, Antonio Di Stasio, Pellegrino Cerino

**Affiliations:** 1 Department of Medicine, Surgery & Dentistry ‘Scuola Medica Salernitana’, University of Salerno, 84081, Baronissi (SA), Italy; 2 Regional Reference Center for Rare Tumors, Department of Oncology & Hematology, AOU Federico II of Naples, 80131, Naples, Italy; 3 Centro di Referenza Nazionale per l'Analisi e Studio di Correlazione tra Ambiente, Animale e Uomo, Istituto Zooprofilattico Sperimentale del Mezzogiorno, 80055, Portici (Na), Italy

**Keywords:** biomonitoring, Land of Fires, study design

According to the WHO, approximately a quarter of deaths recorded worldwide in 2012 were ultimately caused by unhealthy environmental factors, including air, water and soil contamination, indoor and electromagnetic pollution, agricultural methods and occupational exposure [1]. In advanced countries, industrialization is usually one of the main culprits for pollution, which can be interpreted as the downside of economic growth and progress. In the provinces of Naples and Caserta in the Campania region of southern Italy, which encompass a total of 195 municipalities and include grazing and farming lands, one contributing factor to environmental pollution has been illegal waste trafficking – a rare scenario in Europe and other industrialized countries. According to Legambiente, one of the most authoritative nongovernmental Italian organizations for environmental protection [2], an estimated 10 million tons of hazardous waste – including waste from the metallurgy and leather industries, dust from fume filters, paint sludge, liquid waste contaminated with heavy metals, asbestos and polluted land from remediation activities – were either buried underground or abandoned in open air in these areas during the years 1991–2014. Waste that is not buried is usually burned to remove traces; hence the nickname ‘Land of Fires’ (LoF), because thousands of fires have been reported. Over the past decades, its profitability and simplicity have fueled the phenomenon of illegal waste trafficking in the LoF, which has only been partially contained by the numerous legal investigations conducted so far and the multiple *ad hoc* legislative interventions, such as the Law 6/2014 [3]. The threats posed by open-air incineration and burial of hazardous waste have generated fears of contamination in both the resident population and consumers of local food products, causing social tensions and both image and economic damage for the local food producers. From an epidemiology perspective, there is consistent evidence that citizens dwelling in the provinces of Naples and Caserta show some excess risk for all-cause mortality and various types of cancer compared with the regional average [4], especially in municipalities located in the northern part of the Naples province and the southeastern part of the Caserta province, where most clusters of slightly to moderately increased cancer mortality [5] have been identified. These findings have been confirmed by the SENTIERI epidemiological study conducted in the LoF municipalities [6], which showed an overall increased risk of all-cause mortality of 5–10%, with higher increases for certain cancers in certain areas (e.g., bladder cancer in the Naples province [7]). Nevertheless, studies have failed to prove a cause–effect relationship between waste exposure and increased cancer-related and all-cause mortality in the area; the presence of dumping sites was not consistently associated with clusters of increased cancer incidence [8]. While the exact magnitude of the consequences of the LoF phenomenon for human health is yet to be determined, the area’s bad reputation has severely harmed the economy over the years. As an example: in 2014, revenues from one of the typical products of Campania, water-buffalo mozzarella, dropped by 57 million Euros [9].

## Institutional initiatives to tackle the Land of Fires crisis

An inter-ministerial task force was set up in 2014 by the Italian Government in order to guarantee the safety of agricultural production [3]. The Experimental Zooprophylactic Institute of Southern Italy (Istituto Zooprofilattico Sperimentale del Mezzogiorno; IZSM), with headquarters in Portici, Naples, is a public health institution operating within the National Health Service, mainly in the field of hygiene and veterinary public health. Over the past 5 years, the IZSM has promoted several projects and scientific researches in an effort to tackle the LoF phenomenon in cooperation with numerous institutions and experts from various fields, united in a large research team that has included over 40 scientists: veterinary doctors, physicians specialized in medical oncology and infectious disease, agronomists, environmental engineers, molecular biologists, bioinformatics, pharmacologists and biochemists. A National Reference Center for Analysis and Correlation Study of Environment, Animals and Humans (Centro di Referenza Nazionale per l'Analisi e Studio di Correlazione tra Ambiente, Animale e Uomo), with a special focus on human biomonitoring (HBM), was founded in 2019 at the IZSM and was recognized by the Ministry of Health. HBM allows to assess levels of known xenobioticcompounds and their metabolites in human biological matrices (by measuring biomarkers of exposure) as well as to capture the early biological effects associated with exposure to xenobiotic substances (by measuring biomarkers of effect) [10]. While cross-sectional HBM surveys present a number of limitations, including the origin of the contaminants found and the limited number of subjects and contaminants tested, their unique strength lies in providing unequivocal evidence that both exposure to and absorption of contaminants have taken place.

In the context of the LoF – characterized by a powerful mixture of media attention, fears among the indwelling population, economic implications and the actual need for environmental remediations and effective public health interventions – HBM studies have a tremendous potential to provide critical information regarding the levels of exposure of the population to environmental contaminants. The innovations in study design of biomonitoring surveys discussed here are the result of IZSM experience in the field.

## Innovations in study design & procedures of HBM studies

WHO guidelines suggest including at least 120 citizens per population-based group to allow inter-group comparison [11]. In the Campania Region there are 550 municipalities distributed in five different provinces (Naples, Caserta, Avellino, Benevento and Salerno) [12]. Should the study design of a HBM survey planned in Campania use municipalities to define sub-groups the total sample would require at least 60,000 people (120 times 550). On the other hand, the use of provinces does not appear justified, because they are defined according to administrative confines and are sufficiently large to present substantial heterogeneity in the levels of contamination of environmental matrices. Given the unequivocal relationship between the levels of environmental contamination and the results of HBM studies, our three-step approach for geographical stratification and definition of sample size in regional HBM surveys is based on the use of a synthetic indicator of environmental pressure computed at a municipality level, namely the municipality environmental pressure index (MEPI). MEPI should be computed by analyzing available data regarding sources of environmental contamination (legal and illegal dumpsites, industries, vehicular traffic etc.), analytical data on environmental matrices (soil, water and air), as well as migration pathways from contamination sources to potential targets. MEPI computation at a regional level allows identification of a representative subgroup of municipalities, which should include at least 20% of all municipalities in the region. As a second step, a minimum sample size that allows stratification by age and sex should be set; for example, 200 citizens stratified into three age groups (20–30, 31–40 and 41–50) and by sex. Third, municipalities with similar MEPIs can be grouped into clusters. The MEPI-based approach proposed here has the advantage of including a large number of municipalities within the area of the study, while keeping a reasonably low sample size and providing a representative sample of the population exposed at similar environmental pressures, with savings in financial and human resources as well as time. The weaknesses of this approach lie in the use of a single numerical index to describe a complex phenomenon as environmental pressure.

Once clusters representative of various degrees of environmental pressure are identified, the selection of participants should be rigorously based on random recruitment of citizens. In this regard, mass and social media health communication campaigns, public events and the availability of a call center are essential to increase population awareness and participation.

In order to assess the contribution of diet and lifestyle, participants in HBM studies should be carefully assessed for these factors using validated questionnaires (e.g., those used in the European Prospective Investigation into Cancer and Nutrition studies [13]), which can provide a tremendous wealth of information regarding the relationship between diet and exposure to contaminants. Furthermore, participants should undergo a complete medical examination to assess clinical parameters (e.g., arterial pressure, oxygen saturation and weight) and a full medical history should be taken. Exposure biomarkers should include at least heavy metals and dioxin and dioxin-like compounds. As dioxin and dioxin-like compounds require a considerable amount of blood for testing, the use of a biological assay that measures overall biological activity in terms of activation of the aryl hydrocarbon receptor is recommended because it has the advantages of reduced costs and requirement for a smaller blood sample (a few vs >50 ml of whole blood).

Finally, HBM studies should not miss the opportunity to explore biomarkers of early biological effect, such as telomere length, DNA methylation levels, miRNA expression levels, cytokine levels and antioxidant activity levels. Optimal management of individual results (e.g., those showing levels >95th percentile in any biomarker assessed) is unknown and represents an exciting field of research that should also be considered in the design of HBM studies.

In conclusion, HBM studies are powerful tools capable of collecting a wealth of data and providing an opportunity to tackle the increasing threats to human health posed by environmental pollution. Research on study design is a compelling need for advancement in the field.
